# A Novel Model Based on CNN–ViT Fusion and Ensemble Learning for the Automatic Detection of Pes Planus

**DOI:** 10.3390/jcm13164800

**Published:** 2024-08-15

**Authors:** Kamil Doğan, Turab Selçuk, Abdurrahman Yılmaz

**Affiliations:** 1 Radiology Department, Bursa City Hospital, 16110 Bursa, Turkey; dr.kamil.dogan@gmail.com; 2 Department of Electrical and Electronics Engineering, Kahramanmaras Sutcu Imam University, 46200 Kahramanmaraş, Turkey; 3 Department of Orthopaedics and Traumatology, Faculty of Medicine, Hacettepe University, 06100 Ankara, Turkey; doktorayilmaz@gmail.com

**Keywords:** vision transformer, pes planus, ensemble learning

## Abstract

**Background**: Pes planus, commonly known as flatfoot, is a condition in which the medial arch of the foot is abnormally low or absent, leading to the inner part of the foot having less curvature than normal. Symptom recognition and errors in diagnosis are problems encountered in daily practice. Therefore, it is important to improve how a diagnosis is made. With the availability of large datasets, deep neural networks have shown promising capabilities in recognizing foot structures and accurately identifying pes planus. **Methods**: In this study, we developed a novel fusion model by combining the Vgg16 convolutional neural network (CNN) model with the vision transformer ViT-B/16 to enhance the detection of pes planus. This fusion model leverages the strengths of both the CNN and ViT architectures, resulting in improved performance compared to that in reports in the literature. Additionally, ensemble learning techniques were employed to ensure the robustness of the model. **Results**: Through a 10-fold cross-validation, the model demonstrated high sensitivity, specificity, and F1 score values of 97.4%, 96.4%, and 96.8%, respectively. These results highlight the effectiveness of the proposed model in quickly and accurately diagnosing pes planus, making it suitable for deployment in clinics or healthcare centers. **Conclusions**: By facilitating early diagnosis, the model can contribute to the better management of treatment processes, ultimately leading to an improved quality of life for patients.

## 1. Introduction

Pes planus, or flatfoot, is a prevalent condition characterized by the inward collapse of the arch along the inner side of the foot [1,2]. It is commonly categorized into two types: pediatric pes planus and adult-acquired pes planus (AAPP). Pediatric pes planus is also categorized into two groups: flexible pes planus and painful pes planus. The flexible type is typically characterized by hypermobility of the subtalar joint, and the development of the medial arch normally occurs naturally before adulthood [3,4]. However, painful pes planus in children and adolescents is mostly caused by talocalcaneal coalition [5]. AAPP is acknowledged as a source of discomfort and impairment in adulthood, primarily resulting from posterior tibial tendon deficiency (PTTD) [6].

Tarsal coalition is when two or more tarsal bones fuse together incorrectly, which can lead to painful pes planus in adolescents. Tarsal coalitions are classified into fibrous, cartilaginous, and osseous coalitions based on the abnormal bridge morphology [7]. The exact prevalence of tarsal coalition is uncertain; estimates vary from less than 1% to around 1–2% of the population [8].

The prevalence of AAPP ranges from 5% to 15% in the community. Approximately 1 out of every 100 people in the overall population seeks medical attention for the pain resulting from a flat foot. The prevalence of asymptomatic AAPP, which is also quite prevalent, is not fully known [9,10].

PTTD, the most common reason for AAPP, can be attributed to various factors, including age, obesity, ligamentous laxity, and trauma. There is a watershed area behind the medial malleolus that typically experiences degeneration as a result of a sudden alteration in the orientation of the tibialis posterior tendon. PTTD results in a disruption of force balance [4,6,11]. The peroneus brevis tendon causes forefoot abduction, and the Achilles tendon causes a valgus deformity of the ankle. Subsequently, spring tendon weakness ensues, leading to the collapse of the medial arch [12,13]. 

Pes planus can be diagnosed by both a clinical examination and radiographic methods. The primary radiological approach for investigating pes planus is a full-weight lateral foot X-ray in the stance phase [14,15]. The assessment of AAPP involves measurements on lateral weight-bearing X-rays. These measurements include the talo-first metatarsal angle (Meary’s angle), calcaneal pitch angle, and distance between the medial cuneiform and the floor. However, using these parameters can be time-consuming and may lead to inconsistent results between different observers [16].

The potential of computer-based, automated systems for disease detection from X-ray images is extremely promising. There are many studies in the literature on pes planus detection from X-ray images with machine learning. There are two basic approaches to pes planus detection. The first is to determine whether it exists by classifying images. The second is to calculate the angles used to detect pes planus from the patient’s foot X-ray images. Image interpretation using computer-aided systems equipped with artificial intelligence can be more objective, faster, and more accurate than human interpretation [17,18,19]. Therefore, the computer-based automatic detection of pes planus is an important research topic in the literature.

Convolutional Neural Networks (CNNs) are widely used deep learning models for image processing and classification tasks [20,21]. CNNs have achieved great success in the field of image processing and classification and are particularly powerful tools for medical image analysis. Vision Transformers (ViTs) were originally developed for natural language processing (NLP) and have been adapted for image processing tasks [22]. ViTs are a relatively new approach and perform exceptionally well, especially on large datasets.

In this study, a novel fusion model has been created using both CNNs and ViTs for pes planus detection. There is no study in the literature investigating the performance of a Vision Transformer (ViT) for detecting pes planus from X-ray images. The aim of the study is to evaluate the accuracy and reliability of the combined use of these two models in detecting pes planus. Additionally, an ensemble learning method consisting of a support vector machine (SVM) [23], K-Nearest Neighbors (KNNs) [24], and decision trees (DT) [25] was used. Predictions from each model were either voted on or averaged. Ensemble learning enhances the performance, accuracy, robustness, and stability of predictive models, making it a valuable technique in machine learning and data science. The results obtained show that the integration of CNNs and ViTs offers an innovative approach to developing automatic and precise diagnostic tools and provides an effective solution that can assist clinicians in the automatic detection of pes planus in clinical applications.

## 2. Materials and Methods

### 2.1. Dataset

The dataset consists of weight-bearing X-ray images of patients admitted to the Radiology Department of Elazığ Fethi Sekin City Hospital. These patients were undergoing routine pre-military health screenings or were suspected of having flatfoot conditions. All images were captured using a Philips dual detector digital X-ray machine (65 kV, 6.3 mAs) and stored as JPG files. 

**Figure 1:**
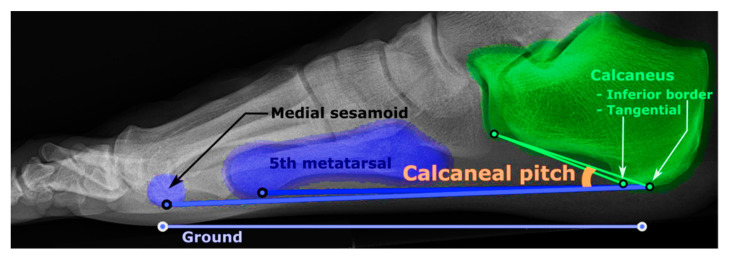
Definition of calcaneal pitch angle [26].

A total of 439 patients provided images. However, X-ray images from 18 of these patients were removed due to low quality. A total of 842 X-ray images from 421 patients were labeled according to the calcaneal angle by two expert radiologists. A third specialist radiologist conducted post-labeling by re-evaluating the conflicting cases. The calcaneal angle is shown in Figure 1. 

If the calcaneal pitch angle was less than 18 degrees, it indicated that pes planus was present, and if it was equal to or greater than 18 degrees, it indicated that pes planus was not present. The distribution of the dataset is given in Table 1.

### 2.2. Image Preprocessing

A large dataset is critical for performance during the training of deep learning models [27]. Otherwise, problems such as overlearning are encountered [28]. When the dataset is limited, it is possible to obtain the desired amount of data by applying data augmentation techniques. First, all images were resized to 224 × 224 dimensions. Ten-degree rotation and mirroring (on the x and y axes) were performed randomly on the images in the dataset. In this way, the number of images was increased from 842 to 1500. Additionally, to avoid data imbalance, 750 images were set as pes planus and 750 images were set as normal. 

### 2.3. CNN and ViT

CNN models have some limitations regarding image classification, especially when global structural information is obtained. Since CNN models tend to better learn a class with a large amount of data, they may make some incorrect classifications [29]. In our study, a model combining a ViT and CNN is presented. This model achieved higher performance than ordinary CNNs in detecting pes planus from ultrasound images. VGG16 was used as the CNN model, and a Swin transformer was used as the ViT. Additionally, classification performance was further improved with ensemble learning consisting of an SVM, K-Nearest Neighbor (KNN), RF, and decision tree classifiers. In addition, it was found that the proposed model performed better than ordinary CNNs regarding data imbalance.

**Figure 2:**
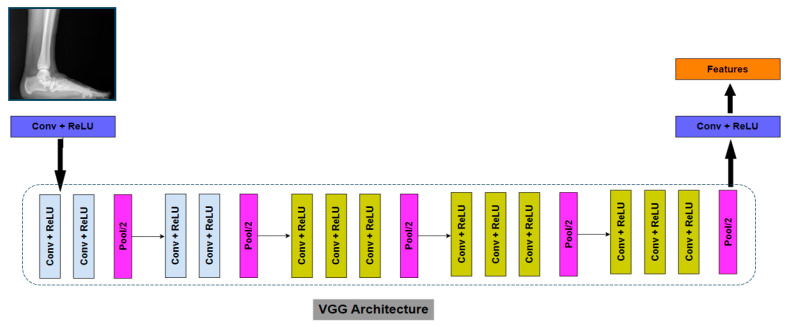
VGG-16-based CNN architecture.

VGG16 consists of 13 convolutional layers (convolutional blocks) in which each block comprises multiple convolutional layers with 3 × 3 filters and a stride of 1 (Figure 2). The number of filters starts at 64 and then doubles after every max-pooling layer. Following the convolutional layers, VGG16 has 3 fully connected layers, also known as dense layers or FC layers, with 4096 neurons each. 

ViT is a transformer architecture originally designed for natural language processing (NLP). In recent years, it has been used for image classification tasks and is a popular architecture with high performance. In a standard ViT architecture, an image is divided into fixed-size segments, which are then linearly embedded and processed by a transformer encoder. However, for tasks that require capturing more complex spatial information or interactions between different parts of the image, multiple transformer blocks or layers can be added to the ViT architecture.

**Figure 3:**
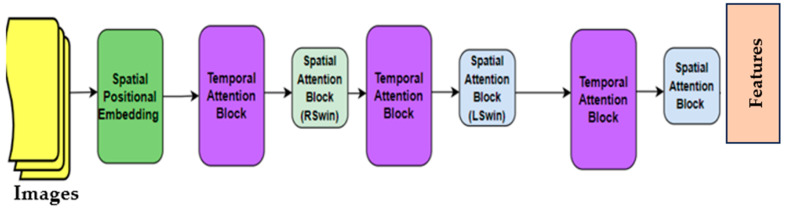
Structure of the ViT model.

For the ViT model, each image was divided into 16 × 16 patches (a total of 196 patches). Then, the location information and class information of each patch were applied as input to the encoder part of the transformer network. Finally, a multilayer perceptron (MLP) was transferred from the transformer network. Since the dataset used was not large, a ViT-B/16-based ViT was chosen. A feature vector length of 768 was obtained from each patch. In total, feature vector dimensions of 196 × 768 were obtained from each image. Figure 3 shows the structure of the ViT model. In the training process, the initial learning rate was set to 0.01. We selected binary cross-entropy loss as our loss function for the binary classification. The momentum and the batch size were set to 0.9 and 64, respectively.

**Figure 4:**
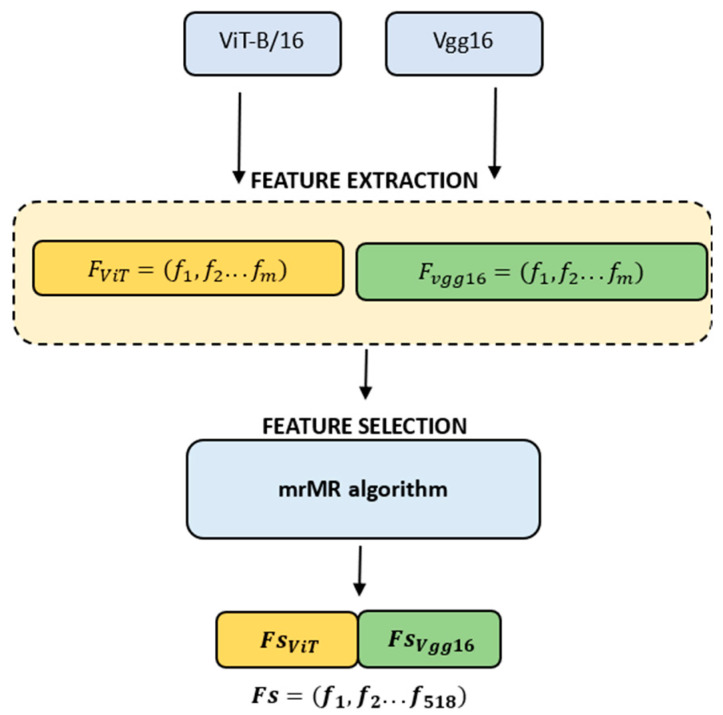
The flow diagram illustrating the proposed model.

Feature selection was performed to eliminate unnecessary features. Among the 13,696 features in the third layer of VGG16, the 32 features with the highest correlation were selected. Similarly, out of the 151,296 features obtained with the ViT, 486 features with high correlation were selected. The mRMR algorithm was used for feature selection. mRMR is a feature selection algorithm based on the Maximum Relevance Minimum Redundancy criterion. It analyzes the relevance between features and the class and the redundancy among the features themselves. For this analysis, Euclidean distance, Pearson correlation, and information measurement are used. The mathematical expressions of the mRMR algorithm are given in Equations (1) and (2) [30]. The proposed model is shown in the flow diagram in Figure 4.

(1) $$Maximum\;Relevance\left(MR\right)=max\left[\frac{1}{\left|F\right|}\underset{f_{i}\in F}{\sum }D\left(f_{i},C\right)\right]$$

(2) $$Minimum\;Redudancy\left(mR\right)=min\left[\frac{1}{{\left|F\right|}^{2}}\underset{f_{i}\in F}{\sum }\underset{f_{j}\in F}{\sum }D\left(f_{i},f_{j}\right)\right]$$

### 2.4. Ensemble Learning

**Figure 5:**
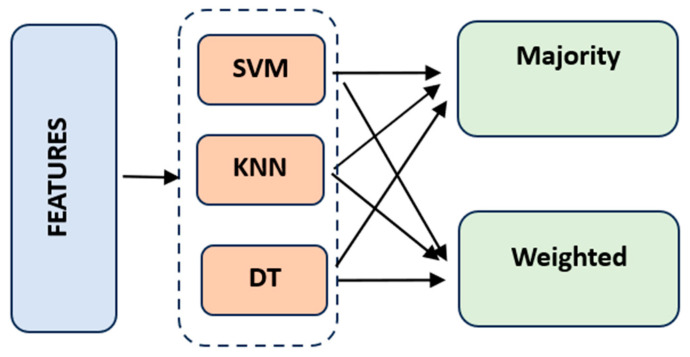
The proposed ensemble learning model.

An ensemble learning-based classification method was applied. Ensemble learning offers better classification performance by combining the most advantageous features of different classifiers. For example, decision trees (DTs) are significantly affected by data change. However, in some cases, they show higher performance than SVMs. Therefore, an ensemble learning combination of an SVM, KNN, and DT was used in the present study. We trained the classifiers with the feature vectors of each image. The proposed ensemble learning model is shown in Figure 5.

Both majority voting and weighted average voting were used. In majority ensemble learning, each model in the ensemble makes its own prediction for a given input or example. The final prediction is then determined by selecting the class label that receives the most “votes” or agreement from the individual models [31]. In classification tasks, the class label with the highest prediction frequency across the ensemble is selected as the final prediction. In a weighted average, each model in the ensemble makes a prediction for a given input. Each prediction is assigned a weight that reflects its relative importance or reliability. The final estimate is obtained by calculating the weighted average of the individual estimates, where each prediction is multiplied by the corresponding weight. 

If we need to express ensemble learning mathematically, the ensemble E contains *n* classifiers, denoted as C_1_, C_2_, …, C_n_ for each classifier *C_t_* (where t is the index of the classifier); its decision regarding class *c_j_* is represented by *d_t,j_*. This can be expressed as the following:

$d_{t,j}=1\;\mathrm{if}\;\mathrm{classifier}\;C_{t}\;\mathrm{predicts}\;c_{j}$
$d_{t,j}=0\;\mathrm{if}\;\mathrm{classifier}\;C_{t}\;\mathrm{does}\;\mathrm{not}\;\mathrm{predict}\;c_{j}\;$

Majority voting and weighted voting are calculated as follows in Equations (3) and (4):

(3) $$\hat{y}=\underset{c_{j}}{\mathrm{argmax}}\overset{n}{\underset{t=1}{\sum }}d_{t,j}$$

(4) $$\hat{y}=\underset{c_{j}}{\mathrm{argmax}}\overset{n}{\underset{t=1}{\sum }}w_{t}d_{t,j}$$

where $\hat{y}$ represents the final prediction and each classifier can be assigned a weight $w_{t}$.

### 2.5. Performance Indices

Sensitivity, specificity, and accuracy values are used to quantify performance. These indices are used for binary classification problems in the literature [32,33]. In addition to the proposed model using the F1 score, individual uses of the Vgg16 and ViT model were also compared. In addition to the performance of different classifiers, the performance of ensemble learning was also examined. In classification problems, accuracy refers to the accuracy rate of a model, as shown in Equation (5). Accuracy is the ratio of correctly predicted samples to the total number of samples. Sensitivity measures how many of the true positives (*TPs*) it correctly identifies, as shown in Equation (6), while specificity measures how many of the true negatives (*TNs)* it correctly identifies (Equation (7)). The F1 score allows for precision and recall values to be taken into account together. In particular, in unbalanced class distributions (i.e., when one class has considerably more or fewer samples than another), the F1 score can be a useful performance metric in addition to accuracy assessment. It is calculated as shown in Equation (8). These metrics are calculated using *TPs, TNs,* false negatives (*FNs*), and false positives (*FPs)*.

(5) $$Accuracy=\frac{TPs+TNs}{TPs+TNs+FPs+FNs}\times 100\%$$

(6) $$Sensitivity=\frac{TPs}{TPs+FNs}\times 100\%$$

(7) $$Specificity=\frac{TNs}{TNs+FPs}\times 100\%$$

(8) $$F1=\frac{2\times TPs}{2\times TPs+FNs+FPs}$$

TPs are the samples that were actually pes planus and were classified correctly; FPs are the samples that were not actually pes planus but were misclassified as pes planus by the model; TNs are the samples that were not actually pes planus but were correctly classified by the model; FNs are the samples that were actually pes planus but were misclassified as not pes planus by the model. 

## 3. Results

In our experiment, a binary classification was used: pes planus and normal. The dataset was divided into 80% for training and 20% for testing. Ten-fold cross-validation was employed to ensure the classification performance. Dropout was used to prevent overfitting. For Vgg16, pretrained weights were initially used. The Adam optimizer was chosen as the optimizer, and 100 epoch models were trained. In Table 2, the performances of the CNN and ViT networks in the fusion model are shown separately.

The fusion model consisting of Vgg16 and ViT performed better than the Vgg16 and ViT models used alone. The accuracy, sensitivity, and specificity values of the proposed fusion model were 96%, 95.2%, and 96.8%, respectively. The lowest performance was achieved with Vgg16. The ViT network outperformed Vgg16 in classification. Both the ViT network and the Vgg16 network exhibited advantages over each other in feature extraction. Therefore, the proposed fusion model showed higher performance by using the advantages of both networks. There was an increase in both the number of correct predictions in positive cases (pes planus) and the number of correct predictions in negative cases (normal). The number of FNs, that is, the samples that were actually pes planus but were mistakenly predicted to be normal, was 19. The number of FPs, that is, the samples that were actually normal but were incorrectly classified as pes planus by the model, was 29.

Different classifiers were trained with the features extracted by the fusion model. The performances of each classifier are shown in Table 3. Ensemble learning was used in the experiment. The decisions of the different classifiers were voted on. Two different votes were possible: majority and weighted. Weighted average voting was obtained through ensemble learning, which was more accurate than the individual decisions of the classifiers.

**Figure 6:**
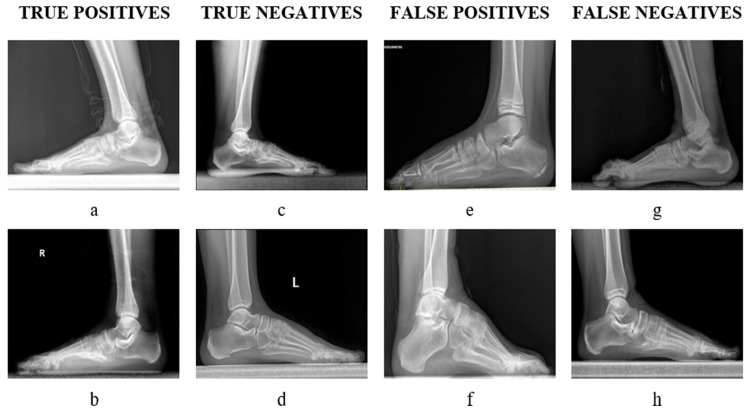
Example images detected as TPs (**a**,**b**), TNs (**c**,**d**), FPs (**e**,**f**), and FNs (**g**,**h**).

In our experiment, the SVM showed a higher performance than the KNN and DT. The lowest performance was achieved with the DT. In community learning, very similar performances were obtained with two different votes. Based on these metrics, EL (weighted) generally outperformed the other classifiers in terms of accuracy, sensitivity, specificity, and the F1 score. However, the choice of classifier ultimately depends on the specific requirements and characteristics of the dataset and the problem at hand. Classified images are shown in Figure 6.

The fusion model developed in this study, consisting of a combination of a CNN and ViT, offers a solution for the automatic detection of pes planus. This model is capable of detecting the presence of pes planus by analyzing features from foot images. Similar studies in the literature generally focus on the detection of pes planus using only one CNN model or more than one CNN model together (Table 4). However, the aim of the present study was to create a more powerful model by combining different architectures such as the CNN and ViT architectures. The results obtained show that the developed fusion model has higher accuracy rates in pes planus detection than the CNN and ViT models used alone. This indicates the model’s ability to better analyze complex structures in the sole of the foot. In addition, due to the generalization ability of the fusion model, reliable results can be obtained in different patient groups and under various conditions. 

## 4. Discussion

The continuing need to address specific clinical problems of the foot with kinematic measurements is pushing research towards the design of new or improved multicompartmental models, as well as expanding the availability of reference data for different pathologies and age groups.

Various methods have been used in the literature for the automatic detection of pes planus. Jian et al. used the Canny edge detection algorithm to determine the arch angle required to detect pes planus. Pes planus was detected according to the principle of obtaining the arch angle by identifying key points. They also used a Gaussian filter to remove noise in images [35]. Image processing-based applications significantly affect image contrast performance. Kao et al. counted the white pixels in an image to identify the calcaneal and fifth metatarsal bones. They determined the arch angle by identifying key points on the calcaneal and fifth metatarsals through binary images. They stated that 73% of all cases were detected correctly [36].

Machine learning algorithms are also used effectively to detect pes planus. Nitris et al. proposed feature extraction based on ResNet50. They suggested a new convolutional neural network (CNN) model that includes Adam optimization. They divided an image into three regions and detected key points separately in each region. A single CNN model’s ability to extract features from an image is limited, which restricts its performance. Gul et al. proposed a new model for the detection of pes planus by combining different CNN models. Image features were extracted with MobilenetV2, which provided the highest performance. Then, dimension reduction was carried out for feature selection. Classification was performed with a support vector machine (SVM), and a 95.14% accuracy was achieved [3].

There could be multiple potential causes of FN and FP outcomes. Initially, the suggested system lacked sufficient training on two groups: adolescent X-rays with open physis and those with other foot abnormalities. A pes cavus deformity (Figure 6f) and an adolescent foot with an open physis (Figure 6e) were most likely to blame for two of the FP results in our test group. Comprehending the cause of FN X-rays is more complicated. We categorized X-rays based on the calcaneal inclination angle, which is determined by measuring the angle produced by lines running from the bottom of the calcaneocuboid joint to the same horizontal line running along the bottom surface of the calcaneus to the lowest point of the fifth metatarsal head [37]. We discovered that, in our FN outcomes, the other metatarsal bones completely encircled the fifth metatarsal bone, making it impossible for our AI system to precisely detect its borders. This may be a potential cause of the FNs.

The proposed model has significant potential for various applications. Firstly, it can be employed by physicians in patient care settings. Lateral weight-bearing foot X-rays are commonly employed to radiographically assess individuals who present to outpatient clinics with suspected pes planus. Our model allows clinicians to make pre-judgments about patients’ pes planus status before taking standard pes planus angle measurements. This approach can expedite patient care. Our developed model could also be applied in the military recruitment process. Several countries enforce mandatory military service for all male citizens. During this procedure, it is necessary to examine them for any potential abnormalities in their feet. Our developed model can assist by evaluating radiographs for a large number of individuals. Moreover, the model can serve as a valuable tool in research. When applied to large datasets of lateral weight-bearing foot X-rays, our model efficiently identifies pes planus.

This study has limitations. We utilized the calcaneal inclination angle in lateral weight-bearing foot X-rays to detect pes planus. However, it is important to note that the underlying causes of pes planus vary. Similar to other orthopedic X-ray assessments, the condition of pes planus should be examined by utilizing a minimum of two X-rays taken from varying angles. In addition, three-dimensional computed tomography (CT) is valuable for the purpose of surgical planning. Future studies may utilize X-rays taken from various angles and CT imaging to enhance the accuracy of detecting pes planus. Another limitation is the restricted quantity of X-rays available. Future studies could focus on further enhancing the model’s performance by utilizing larger and more diverse datasets to reduce FPs and FNs in the adolescent and open-physis groups, particularly. The last limitation is that the F1 score of our proposed model under data-imbalance conditions was not investigated. 

In conclusion, the fusion model developed for automatic pes planus detection yielded promising results and could play a significant role in both academic research and clinical practice, contributing to the early diagnosis and treatment of pes planus.

## 5. Conclusions

The results obtained in this study indicate that our fusion model, which integrates a CNN and ViT, has promising potential for the automatic detection of pes planus. The success of the model is evident in its high accuracy rates and sensitivity values. Furthermore, it outperformed previous approaches in pes planus detection, offering a new perspective in the field and potentially serving as a valuable tool in clinical applications. 

## Figures and Tables

**Table 1:** Distribution of the dataset.

	Category	Pes Planus		Normal	
		Patient (*n*)	Image (*n*)	Patient (*n*)	Image (*n*)
Age	14–24	94	188	98	196
	25–35	78	156	88	176
	35–47	29	58	34	68
Sex	Male	171	342	180	360
	Female	30	60	40	80

**Table 2:** Comparison of the fusion model with ViT and Vgg16.

Model	Accuracy (%)	Sensitivity (%)	Specificity (%)	F1 Score
Vgg16	90	89.8	91.2	91.5
Vit-B/16	94.2	93.9	94.2	94.1
Vgg16-ViT	96.8	97.4	96.8	96.1

**Table 3:** Performances of different classifiers and ensemble learning.

Classifier	Accuracy (%)	Sensitivity (%)	Specificity (%)	F1 Score
SVM	94	93.3	94.7	94.1
KNN	92.3	91.8	93.4	92.9
DT	90.2	89.48	90.8	90.3
EL (majority)	95.8	95	96.4	95.9
EL (weighted)	96.8	97.4	96.3	96.8

**Table 4:** Similar studies detecting pesplanus based on CNN.

Study	Method	Accuracy	Sensitivity	F1 Score
Alsaidi et al. [34]	CNN/Random Forest	88.75	88.00	85.71
Gul et al. [3]	Pyramid MobilenetV2	95.14	96.86	95.22
Proposed	Fusion Model (CNN + ViT)	96.8	97.4	96.8

## Data Availability

The dataset is available at https://www.kaggle.com/datasets/suleyman32/pesplanus-two-class-dataset (accessed on 8 August 2024).
